# Tracking WNV transmission with a combined dog and wild boar surveillance system

**DOI:** 10.1038/s41598-025-89561-5

**Published:** 2025-04-01

**Authors:** Cora M. Holicki, Ute Ziegler, Wolfgang Gaede, Kerstin Albrecht, Jana Hänske, Jörg Walraph, Balal Sadeghi, Martin H. Groschup, Martin Eiden

**Affiliations:** 1 Institute of Novel and Emerging Infectious Diseases, Friedrich-Loeffler-Institut, Federal Research Institute for Animal Health, Südufer 10, 17493 Greifswald-Insel Riems, Germany; 2 Department of Veterinary Medicine, State Office for Consumer Protection Saxony-Anhalt, Haferbreiter Weg 132, 39576 Stendal, Germany; 3 Department of Veterinary Medicine, Saxon State Laboratory of Health and Veterinary Affairs, Jägerstraße 8/10, 01099 Dresden, Germany; 4 Department of Veterinary Medicine, Saxon State Laboratory of Health and Veterinary Affairs, Zschopauer Straße 87, 09111 Chemnitz, Germany; 5 Present Address: Viroscience, Erasmus Medical Center, Rotterdam, the Netherlands

**Keywords:** Virology, Viral epidemiology, Viral reservoirs, West nile virus

## Abstract

West Nile virus (WNV) is a mosquito-borne flavivirus mainly circulating in eastern Germany, causing annually reoccurring epizootics in the avifauna as well as sporadic infections in humans and horses. WNV is closely-related to Usutu virus (USUV) and tick-borne encephalitis virus (TBEV) and co-infections thereof are becoming more frequent. To not solely be dependent on the monitoring of wild birds and horses the availability of alternative sentinel species is advantageous. The study examined the seroprevalence of WNV antibodies (Abs) in eastern Germany in readily available species: dogs, wild boars, sheep, and goats. An NS1-ELISA was implemented to ease future differentiation of cross-reacting flavivirus Abs with a sensitivity of 92.3 and 90.9% for dog and wild boar sera, respectively. Flavivirus seroprevalences were the highest in wild boars with 42.03%, followed by dogs with 7.86%, and small ruminants with 1.57%. In the wild boars and dogs, WNV Abs were most frequent (17.64 and 3.90%, respectively) while seroprevalences in small ruminants and of USUV were lower. The NS1-ELISA is cost-efficient and reliable in monitoring WNV Abs in dogs as well as wild boars and the combined testing thereof could be ideal in detecting semi-urban transmission events prior to wildlife-human spill overs.

**Supplementary Information:**

The online version contains supplementary material available at 10.1038/s41598-025-89561-5.

## Introduction

West Nile virus (WNV; *Flaviviridae*; *Flavivirus*) is a zoonotic virus that is maintained in a sylvatic mosquito-bird transmission cycle with spill over infections to incidental hosts including horses and humans. Even though the majority of human infections are asymptomatic, patients can develop flu-like symptoms (WNF: West Nile fever) or even a severe neuroinvasive disease (WNND: West Nile neuroinvasive disease) in 1% of the cases^1^. Likewise, in horses most infections are subclinical but 10% of them can develop a clinical illness with signs ranging from unspecific lethargy and fever to neurological disease^2^. The virus has a very broad vector and host range, replicating in several mosquito species and infecting numerous mammals as well as reptiles and amphibians^3^.

With its origin in the West Nile district of Uganda in 1937^4^, the virus is to date present almost worldwide with epidemics/epizootics typically occurring annually from summer until fall. With the first detection of WNV in East Germany in 2018^5^, the virus not only became a focus point for veterinary research but also for the public health sector of the country. Surveillance efforts in Germany in the past have focused primarily on captive and wild bird species^5–9^, and have been instrumental for the implementation of public health measures (e.g., blood donor surveillance^10^ and mosquito testing^11^). By their means WNV, as well as the closely related flavivirus Usutu virus (USUV), were isolated for the first time from bird^5,12^ as well as mosquito samples^11–13^. They also enabled the tracing of WNV and USUV expansion in Germany with USUV, since 2018, occurring throughout all German federal states^8,9^ and WNV gradually spreading from the east west- and southward^5–7^. Nonetheless, the surveillance of live/dead and wild/captive birds has its limitations, including particularly small sample volumes. The success of such studies depends greatly on an excellent cooperation between state veterinary services, bird clinics, wild bird rescue stations, zoos, wild parks, and academic institutes. One alternative strategy would be to sample horse sera yet this is also beginning to face difficulties with an increasing number of vaccinated horses against WNV in eastern Germany. The Standing Commission on Vaccination in Veterinary Medicine (Ständige Impfkommission Veterinärmedizin; StIKo Vet) recommends the vaccination of horses kept in or traveling to WNV-endemic regions^14^. Fortunately, due to the wide host range of WNV, the usage of alternative sentinels including pets as well as wild mammals is feasible and can especially help in detecting WNV-transmissions outside of the enzootic bird-mosquito-bird cycle^15^. Especially, the surveillance of wild mammals can span versatile habitats and time frames in which viral transmissions may take place^16^.

Eurasian wild boars, for example, are hunted as part of management programs in Germany, especially since the introduction of African swine fever from Poland in 2020^17^, making blood samples easily accessible (high sample number and volume) without having to face extra costs^15^. This is in comparison to the labor-intensive sampling of live wild birds performed among others for the purpose of monitoring bird-transmitted viruses including flaviviruses. Furthermore, wild boars are ideal sentinels as they are highly likely to come in-contact with infected mosquitoes due to habitat preferences for forest landscapes^18^, physical traits (e.g., low hair density and thin skin epidermis^19^), and vector-host feeding preferences^20^. Furthermore, pigs in general are known to show a strong seroconversion against certain viruses including WNV^21^. As omnivorous scavengers wild boars may also become infected with WNV due to a possible oral transmission, as already shown for cats and dogs^22^ but refuted for domestic pigs^21^. A herd can span a wide geographical range utilizing diverse environmental habitats including buffer zones near woodlands (e.g., clear-cuts and deciduous forests), swamps, brushlands, and crop fields^23^. As such they often live in close contact to human settlements, thereby not only destroying agricultural crops but also posing a source for wildlife-human viral transmissions (e.g., Aujeszky’s Disease, Hepatitis E, Japanese encephalitis virus, Influenza virus, or Nipah virus)^24^. Lastly, it is hypothesized that the pathogenesis of WNV in wild boars is similarly weak to that in domestic pigs^21^ and as such wild boars probably do not constitute as amplifying hosts.

Further candidate sentinel species for WNV are domestic species such as dogs and small ruminants (sheep and goats). Similar to the accessibility of wild boar samples, sheep and goat sera can readily be obtained from regional State Veterinary Investigation Centers in the frame of other disease monitoring programs. Likewise, excess dog sera are available at private veterinary diagnostic laboratories. Dogs and small ruminants are also exposed to vectors over an extensive period of time (outdoor activities or grazing in meadows, respectively), yet in contrast to wild boars, their movements are either geographically restricted or can be readily traced^25^. Due to their housing with or in the vicinity of humans they can be a sensitive indicator for the risk humans may face from arboviruses like WNV. Dogs, sheep, and goats are probably not a source of infection for humans, as they are dead-end-hosts for WNV, and do not develop viremia after an infection or only very low levels without clinical signs^22^. Nonetheless, individual cases of severe infections (encephalitis/meningoencephalitis) have been described for dogs^26–28^ and sheep^29–31^. In Europe, several studies in the past have analyzed dog sera (from pet/hunting/military dogs) for zoonotic flavivirus antibody prevalences with varying results (summarized by García-Bocanegra^32^): Denmark (30%;^33^), France (9.1–12.5%^34^), Spain (4.8%^35^). In addition, specific WNV seroprevalences were determined in Belgium (0%^36^), in France (6.7–8.4%^34,37^), in Italy (55.5%^38^), in Spain (1.3–1.6%^35^), and in Turkey (37.7%^39^). By contrast, seroprevalence studies in small ruminants are scarce and prevalences very low: e.g., 1% for WNV in Turkey^39^ and 0% in France^37^.

The here described study successfully examines the seroprevalence of WNV and of the closely-related USUV and tick-borne encephalitis virus (TBEV) in wild boar, dog, and small ruminant sera from eastern Germany. It verifies that these species, especially wild boars and dogs, are ideal sentinels for zoonotic flaviviruses in rural as well as urban areas due to their accessibility, their site fidelity and habitat diversity, and their close contact to humans.

## Materials and methods

### Samples

**Fig. 1:**
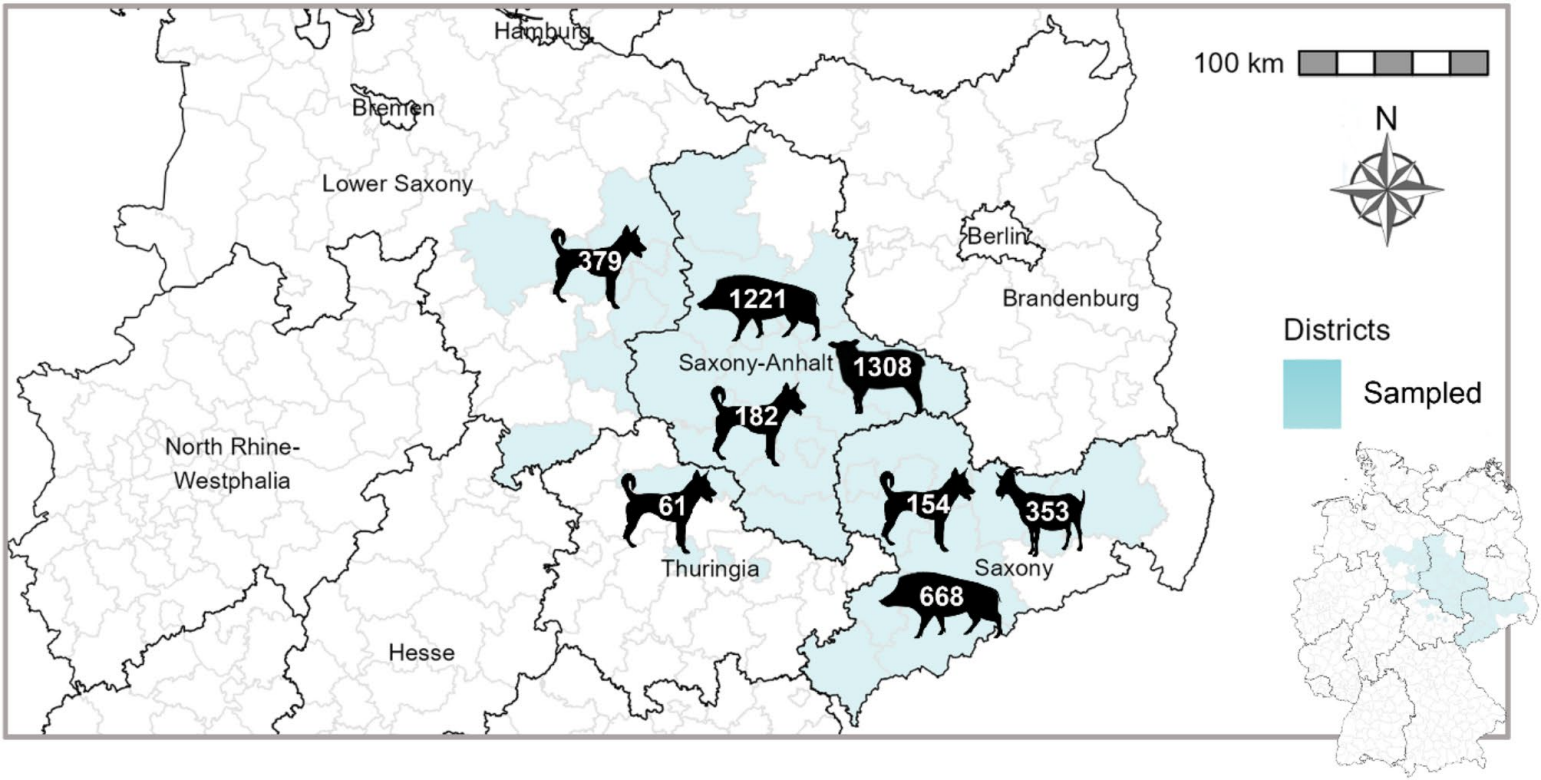
Map of sampling area in East and Central Germany: Districts in Saxony, Saxony-Anhalt, Thuringia, and Lower Saxony from which samples were collected are portrayed in light blue. Species icons display the species from which the samples originated and the number thereof per federal state. Map was created with GADM version 4.1. (https://gadm.org).

The districts from which samples originated, mainly belonging to East and Central Germany, are displayed in Fig. 1. Blood sera from wild boar (*n* = 1889) and small ruminant sera (*n* = 1661) were collected from two federal states in eastern Germany (Saxony and Saxony-Anhalt), from districts known to have been exposed to WNV (risk areas) at the time when the study was initiated as well as from neighboring districts. Dog sera (*n* = 776) were collected from four different federal states (Saxony; SN, Saxony-Anhalt; ST, Thuringia; TH, and Lower Saxony; NI) covering cities as well as rural districts. The majority of the samples were collected from 2020 to 2022, with an additional 33 sheep sera from 2018. The wild boars were sampled by hunters and the blood was collected from the thoracic cavities or by heart puncture. The sheep were sampled by local veterinarians in the frame of herd investigations. Sera were sent to the State Veterinary Investigation Offices for routine diagnostics and shipped frozen to the Friedrich-Loeffler-Institut (FLI). Similarly, surplus serum samples from dogs were obtained on ice from the private veterinary laboratory Laboklin GmbH & Co. KG (Bad Kissingen, Germany).

### Molecular investigation

Viral RNA from the sera was extracted with the BioSprint 96 (QIAGEN, Hilden, Germany) using the Kit Nucleo^®^Mag VET (Macherey-Nagel, Düren, Germany), according to the manufacturer’s instructions. A one-step Pan-flavivirus Reverse transcription quantitative real-time PCR (RT-qPCR) assay based on SYBR Green melting curve analysis using the QuantiTect SYBR Green RT-PCR Kit (QIAGEN) was performed to test for flavivirus genome contents^40^. PCR products displaying melting curves close to those of the positive controls were sent to Eurofins for Sanger sequencing and were subsequently analyzed by means of the Basic Local Alignment Search Tool (BLAST)^41^ (https://blast.ncbi.nlm.nih.gov/Blast.cgi, NCBI, Bethesda, USA) to identify the origin of the detected genome. Phylogenetic analysis of the two TBEV-partial sequences was done with Geneious Tree Builder using Neighbor-Joining analysis and genetic distances were calculated using the Tamura-Nei Method (Geneious Prime 2021.0.1; https://www.geneious.com). Bootstrap values < 80 are displayed at the nodes. Phylogenetic analysis was carried out with a 182-nt fragment of the NS5 protein targeting position 9082–9264 (according to TBEV isolate Genbank: TEU39292).

### Serological investigation

#### Commercial competition enzyme-linked immunosorbent assay (ELISA)

All sera from the dogs, wild boar, and small ruminants were heat-inactivated and pre-screened using a species-independent commercially available competition enzyme-linked immunosorbent assay (ELISA) for the detection of pan-flavivirus antibodies against the envelope protein following manufacturer’s instructions (ID Screen^®^ West Nile Competition, IDVet, Montpellier, France). Doubtful results were defined by the manufacturer’s specifications. To quantify and differentiate between WNV, USUV, and TBEV specific antibodies, positive samples were either tested in virus neutralization tests (VNTs) and/or in an in-house ELISA based on the non-structural protein 1 (NS1) of WNV, USUV, or TBEV.

#### Virus neutralization test (VNT)

The VNT for WNV was completed under biosafety level (BSL) 3 conditions using Vero B4 cells and the WNV strain Germany (lineage 2, GenBank accession no. MH924836). To exclude cross-reacting neutralizing antibodies (nAbs) against USUV and TBEV a VNT on Vero 76 cells using USUV strain Germany (Europa 3, GenBank accession no. HE599647) was performed as well as with PK-15 cells using the TBEV strain Neudoerfl (GenBank accession no. U27495; kindly provided by G. Dobler, Bundeswehr Institute of Microbiology, Munich, Germany). All used cells derived from the collection of Cell Lines in Veterinary Medicine (Friedrich-Loeffler-Institut (FLI), Germany). VNTs were performed according to standard protocols as described in several previous studies^42^ with minor modifications. As positive controls, WNV-, USUV-, or TBEV-positive sera (from animal infection or vaccination studies) with known antibody titers were included in each VNT, respectively, in addition to negative control sera. The 50% neutralization dose (ND_50_) is the reciprocal of the serum dilution at which 50% of the cytopathogenic effect was inhibited and was calculated according to the Behrens-Kaerber method^43^. Samples were considered positive when ND_50_ was 10 or higher and negative when lower than 10. Sera was considered specific for one of the three flaviviruses when only one of the viruses was neutralized or the corresponding titer against one was fourfold higher than that against the other flaviviruses^44^. Sera samples that could, however, not be differentiated due to similar antibody levels against two or all three of the flaviviruses are only described as having flavivirus nAbs. Explanations for these findings could be an infection by an undetermined flavivirus or multiple infections with different flaviviruses.

#### In-house Indirect NS1-ELISA

An in-house indirect ELISA based on the NS1 protein was established in the frame of this study for WNV, USUV, and TBEV. Recombinant NS1 proteins of the three different flaviviruses were ordered from The Native Antigen Company (Kidlington, United Kingdom) and delivered in the following concentrations: WNV (0.78 mg/ml), USUV (0.49 mg/ml), and TBEV (0.53 mg/ml). The ELISA was then performed as follows: Invitrogen™ Nunc MaxiSorp™ flat-bottom immunoplates (Thermo Fisher Scientific, Dreieich, Germany), were coated with 25 ng WNV-/USUV-/TBEV-NS1 per well (diluted accordingly in 100 µL 0.6 M carbonate-bicarbonate buffer pH 9.6) and incubated overnight at 4 °C. Plates were washed three times with 300 µL/well of washing buffer (Phosphate-buffered saline (PBS)/0.1% Tween 20). They were subsequently blocked with 200 µL/well of blocking solution (0.1% gelatin diluted in PBS) and incubated for 1 h at 37 °C. Plates were washed as previously described. 100 µL/well of the diluted sera samples or control sera (diluted 1:100 in the dilution buffer: 1% rabbit serum diluted in PBS/0.1% Tween 20) were added to the plates and they were again incubated for 1 h at 37 °C. Plates were washed as before and horseradish peroxidase (HRP, Hersteller fehlt) conjugated host-specific secondary antibodies diluted in dilution buffer were added (100 µL/well). The following antibodies and dilutions were used: for dog sera a goat anti-dog antibody (1:1000; Sigma-Aldrich, Taufkirchen, Germany), for wild boar and goat sera a protein A/G antibody (1:30000; Thermo Fisher Scientific), and for sheep sera a donkey anti-sheep antibody (1:10000; Sigma-Aldrich). The plates were again incubated for 1 h at 37 °C. Lastly, plates were washed as before, 100 µL/well of 2.20-azinodiethylbenzothiazoline sulfonic acid substrate (ABTS, Roche, Mannheim, Germany) was added, and the plates were incubated for 30 min at room temperature in the dark. Substrate-binding was stopped by adding 50 µL 0.5 M sodium dodecyl sulphate. OD-values were measured at 405 nm using a Tecan plate reader Infinite 200 PRO (Tecan, Männedorf, Switzerland). The ELISA results were compared to that of the VNTs (diagnostic gold standard) and receiver operating characteristic (ROC) analyzes were performed with regard to the criterion “Maximization of sensitivity and specificity” to calculate cut-offs, sensitivity, and specificity.

### Statistical analysis

Data visualization, analysis, and statistical computing (95% confidence intervals, chi-squared tests, and pairwise Wilcoxon rank sum tests) was conducted in R (v4.2.2, x64)/R Studio (Version 2022.12.0 + 353)^45,46^ using the following additional packages: readxl^47^, raster^48^, dplyr^49^, sf^50,51^, ggplot2^52^, ggthemes^53^, scales^54^, ggsignif^55^, and Hmsic^56^. F Freely available animal silhouettes were downloaded from https://www.phylopic.org and editing of the figures was performed using Adobe Photoshop CS5 (Version 12). The stacked bar charts from Supplementary Figure S1 were created using Microsoft Office Professional Plus Excel 2019. To determine the optimal cut-off for classifying the NS1-ELISA results as positive or negative, ROC curve analysis was performed using MedCalc software (version 19.6, MedCalc Software Ltd, Ostend, Belgium; https://www.medcalc.org; 2020). The optimal cut-off was set as the point on the ROC curve that yielded the highest Youden’s index value within the model’s output.

### Ethics statement

No experiments on vertebrates were performed as part of this study. Serum samples obtained for diagnostic purposes were solely retrospectively and anonymously analyzed. The ARRIVE guidelines therefore do not apply to this study. Residual serum samples from routine diagnostics of shot wild boars and small ruminants were available from the regional State Veterinary Investigation Centers. Additionally, residual serum samples from clinical investigations of dogs were obtained from the private veterinary laboratory Laboklin GmbH & Co. KG (Bad Kissingen, Germany). Sampling of live animals was solely done by trained veterinarians and in accordance with national and European legislation (EU council directive 86/609/EEC for the protection of animals). Sampling of shot wild boars was carried out in accordance with the applicable legal regulations (§ 28a Bundesjagdgesetz (Federal Hunting Act, BJagdG) and § 40e Bundesnaturschutzgesetz (Federal Nature Conservation Act, BNatSchG)).

## Results

For the study the following serum samples were tested: 776 from dogs, 1889 from wild boar, and 1661 from small ruminants (sheep and goats). Dog sera was collected from four federal states in East and Central Germany (Saxony-Anhalt (ST), Saxony (SN), Thuringia (TH), and Lower Saxony (NI)), while the collection of wild boar and small ruminant sera was limited to ST and SN, focusing on district rather than federal state level. Samples from 2018 to 2022 were examined.

### Molecular results

**Fig. 2:**
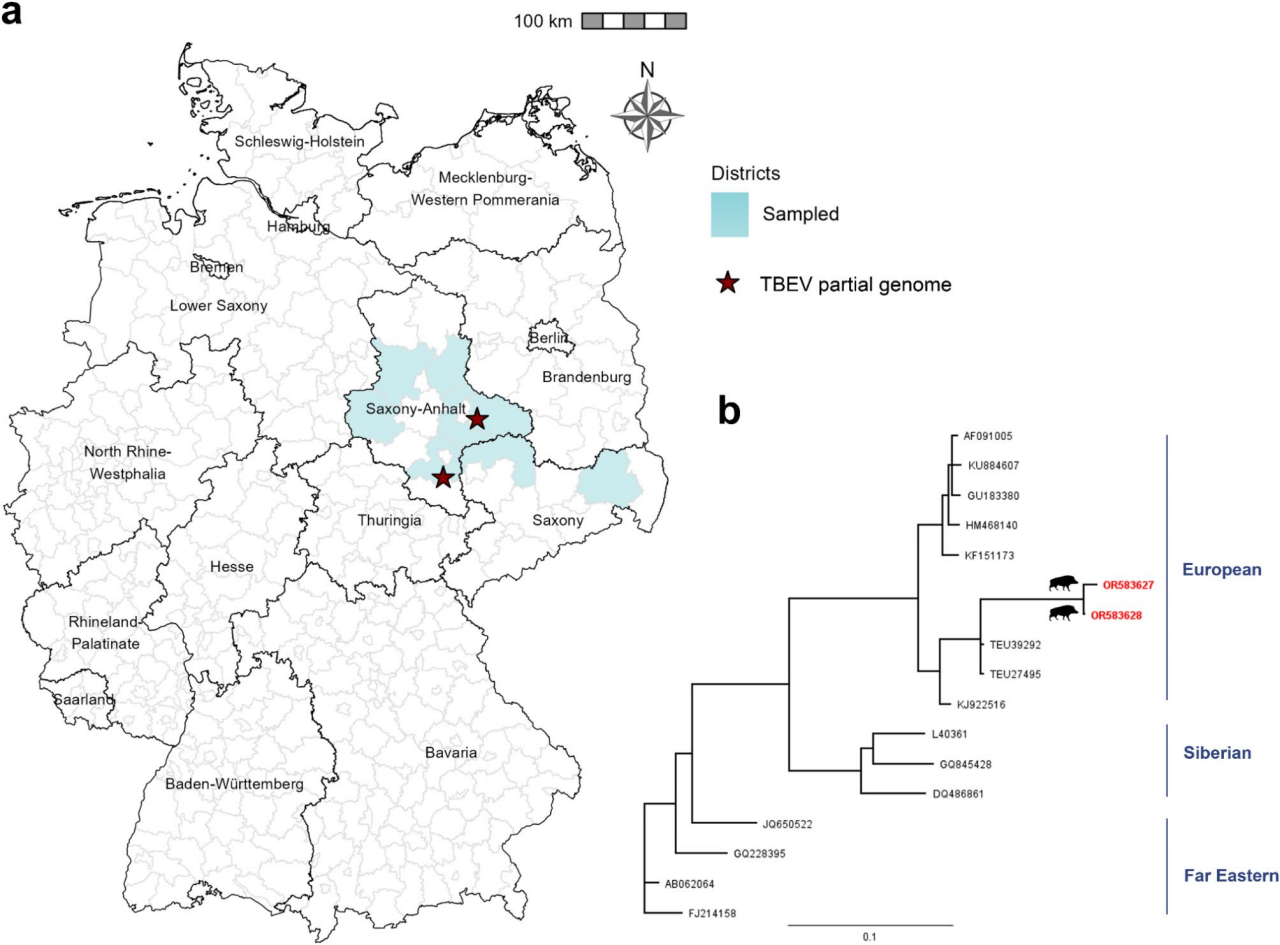
Molecular detection of partial genomes from tick-borne encephalitis virus (TBEV) in wild boars from eastern Germany. (**a**) Map showing the origin of the two TBEV-positive serum samples (indicated by a red star) from two districts of the federal state of Saxony-Anhalt. Created with R (v4.2.2, x64)/R Studio (Version 2022.12.0 + 353)^45,46^. (**b**) Phylogenetic tree of whole genome sequences available on GenBank and the partial NS5 sequences obtained from the two TBEV isolates obtained in this study (GenBank Accession nos. marked in red). Created with Geneious Prime 2021.0.1. Map was created with GADM version 4.1. (https://gadm.org).

The majority of the samples (4.138/4.326; 95.65%) were screened by pan-RT-qPCR for flavivirus genomes. TBEV-genome sequences were detected in two wild boar samples (2/1873) from eastern Germany (Fig. 2). Both of the TBEV-positive samples were collected in ST: one in December 2020 in the district of Saalekreis (wild boar WS/20/58, GenBank Accession no.: OR583627) and the other in January 2021 in the district Wittenberg (wild boar WS/21/83, GenBank Accession no. OR583628). The two TBEV isolates appear to cluster within the European subtype. Neither the WNV nor USUV genome could be detected in the tested wild boar (1873/1889), dog (648/776), or small ruminant sera (1617/1661).

### Serological results

The raw data from all serological tests (pan-flavivirus-ELISA, VNTs, and NS1-ELISAs) are listed in Supplementary Tables S1–S4.

#### ELISA

The results from the ELISA analyzes (i.e., pan-flavivirus-seroprevalences and statistical evaluations) are depicted for all tested species (dogs, wild boar, sheep, and goats) in Table 1 or for each individual sample in Supplementary Tables S1–S4.

Of the examined dogs (*n* = 776) 7.86% (95% CI 6.07–9.98) had flavivirus-specific antibodies, with higher seroprevalences in SN (12.34%; 95% CI 7.59–18.59) and ST (10.44%; 95% CI 6.40–15.82) than in NI (5.54%; 95% CI 3.46–8.35) and TH (3.28%; 95% CI 0.40–11.35). The variance across the examined federal states is statistically significant (Pearson’s chi-squared test: *p <* 0.05*). There, however, appears not to be a sex or age dependency in the seroprevalences of flavivirus antibodies in dogs.

By contrast, 42.03% (95% CI 39.79–44.30) of the wild boar sera (*n* = 1889) had flavivirus-specific antibodies, with a statistically significant (chi-squared test: *p* < 0.001***) higher seroprevalence in North and East SN (district Bautzen) than the examined districts in ST (50%;95% CI 46.14–53.86 and 37.67%; 95% CI 38.14–43.98, respectively). This was also the case on district level in ST (chi-squared test: *p* < 0.001***), with the lowest seroprevalence found in the district Harz (8.66%; 95% CI 5.96–12.07) and the highest in the district Anhalt-Bitterfeld (56.15%; 95% CI 48.72–63.38).

Only very low flavivirus-seroprevalences (1.57%; 95% CI 1.03–2.29) were observed in the examined small ruminants (sheep; *n* = 1308 and goats; *n* = 353). The majority of the samples, originating from sheep from ST, had slightly higher seroprevalences (1.83%; 95% CI 1.18–2.72) than the goats from SN (0.57%; 95% CI 0.07–2.03). However, the reliability of the results for the goats is limited as only 353 animals were sampled from two herds in the north- and southeast of Leipzig in SN. As the samples were tested either individually or as pools of approximately five animals a statistical evaluation cannot performed with these results.

**Table 1:** Serological results for dog, wild boar, and small ruminant sera tested by a pan-flavivirus competitive ELISA.

Dog						
Factor	No. of samples	Flavivirus Ab Pos.	Flavivirus Ab Dbt.*	Flavivirus Ab Neg.	% Pos. (95% CI)	*p* of data set
Total dogs	776	61	0	715	7.86 (6.07–9.98)	
Year						
2021	639	54	0	585	8.45 (6.41–10.88)	X-squared = 1.7388, df = 1, *p*-value = 0.1873
2022	137	7	0	130	5.11 (2.08–10.24)	
Gender						
Male	368	26	0	342	7.07 (4.67–10.18)	X-squared = 5.1528, df = 2, *p*-value = 0.07605
Female	367	28	0	339	7.63 (5.13–10.84)	
n.s.	41	7	0	34	17.07 (7.15–32.06)	
Age						
< 1	11	0	0	11	0.00 (0.00–28.49)	X-squared = 9.6323, df = 7, *p*-value = 0.2104
1–3	123	15	0	108	12.20 (6.99–19.32)	
4–6	123	12	0	111	9.76 (5.14–16.42)	
7–9	148	13	0	135	8.78 (4.76–14.55)	
10–12	192	9	0	183	4.69 (2.17–8.71)	
13–15	101	9	0	92	8.91 (4.16–16.24)	
> 15	16	1	0	15	6.25 (0.16–30.23)	
n.s.	62	2	0	60	3.23 (0.39–11.17)	
Federal states						
Saxony-Anhalt	182	19	0	163	10.44 (6.40–15.82)	X-squared = 10.517, df = 3,*p*-value = 0.01465*
Saxony	154	19	0	135	12.34 (7.59–18.59)	
Thuringia	61	2	0	59	3.28 (0.40–11.35)	
Lower Saxony	379	21	0	358	5.54 (3.46–8.35)	

Wild boar						
Total wild boars	1889	794	14	1085	42.03 (39.79–44.30)	
Year						
2020	86	35	2	49	40.7 (30.22–51.83)	X-squared = 3.0913, df = 2, *p*-value = 0.2132
2021	1803	759	12	1032	42.1 (39.80–44.41)	
Federal states						
Saxony-Anhalt	1221	460	11	750	37.67 (38.14–43.98)	X-squared = 27.434, df = 2, *p*-value = 1.103e–06***
Saxony	668	334	3	331	50.00 (46.14–53.86)	
Districts of Saxony-Anhalt						
Saalekreis	183	116	1	66	63.39 (55.96–70.37)	X-squared = 218.21, df = 10, *p*-value < 2.2e–16***
Wittenberg	304	126	2	176	41.45 (35.85–47.21)	
Anhalt-Bitterfeld	187	105	3	79	56.15 (48.72–63.38)	
Börde	86	37	2	47	43.02 (32.39–54.15)	
Harz	358	31	2	325	8.66 (5.96–12.07)	
Jerichower Land	103	45	1	57	43.69 (33.94–53.82)	
Districts of Saxony						
Nordsachsen	288	147	1	140	51.04 (45.11–56.95)	X-squared = 0.31711, df = 2, *p*-value = 0.8534
Bautzen	380	187	2	191	49.21 (44.08–54.36)	

Small ruminant						
Total small ruminants	1661	26	4	1629	1.57 (1.03–2.29)	No statistical analysis performed as sample pools as well as individual samples were analyzed
Year						
2018	33	0	0	33	0.00 (0.00–10.58)	
2020	23	1	1	21	4.35 (0.11–21.95)	
2021	1605	25	5	1575	1.56 (1.01–2.29)	
Federal states						
Saxony-Anhalt (sheep)	1308	24	4	1280	1.83 (1.18–2.72)	
Saxony (goats)	353	2	2	349	0.57 (0.07–2.03)	
Districts Saxony-Anhalt (sheep)						
Saalekreis	230	15	3	212	6.52 (3.70–10.53)	
Wittenberg	209	7	1	206	3.35 (1.36–6.78)	
Anhalt-Bitterfeld	404	1	0	403	0.25 (0.01–1.37)	
Börde	163	0	0	158	0.00 (0.00–2.24)	
Harz	161	0	0	161	0.00 (0.00–2.27)	
Jerichower Land	108	1	0	107	0.93 (0.02–5.05)	
n.s.	33	0	0	33	0.00 (0.00–10.58)	
Herds in district Nordsachsen (Saxony) (goats)						
“Dreiheide”	320	1	2	317	0.31 (0.01–1.73)	
“Mügeln”	33	1	0	32	3.03 (0.08–15.76)	

*No.* number, *Ab* antibodies, *Pos.* positive, *Neg.* negative, *Dbt.* doubtful, *CI* confidence interval, *n.s.* not specified.

#### VNTs

**Fig. 3:**
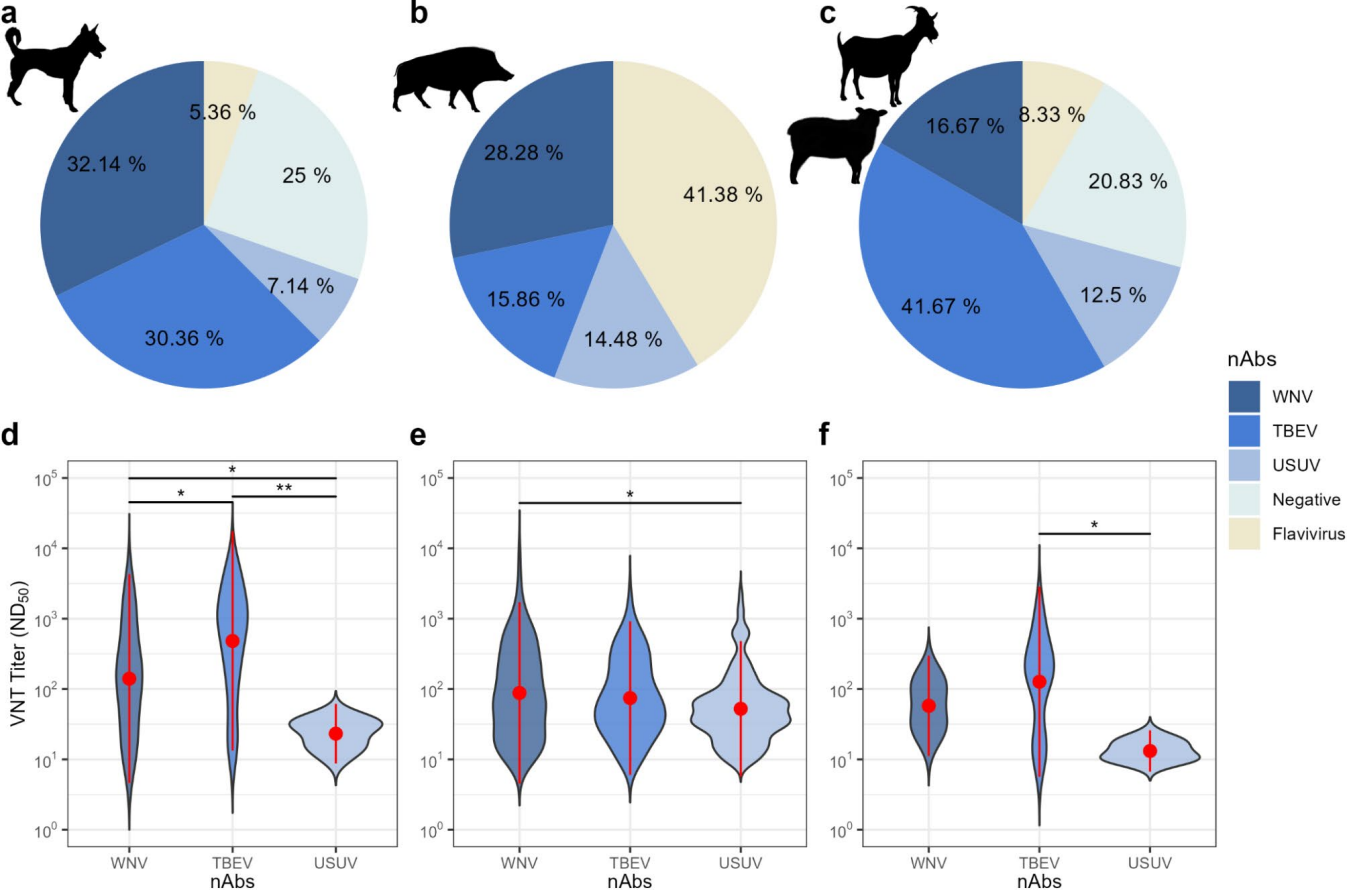
Pie charts (**a**–**c**) illustrating the distribution of the neutralizing antibodies (nAbs) based on the VNTs (Supplementary Table S5) as well as the violin plots (**d**–**f**) illustrating the antibody titer distributions for the three tested flaviviruses (West Nile virus: WNV, tick-borne encephalitis virus: TBEV, and Usutu virus: USUV) for dogs (**a**, **d**), wild boars (**b**, **e**), and small ruminants (**c**, **f**). The red dots in the violin plots denote the mean VNT titers and the red lines the standard deviation thereof. Statistical analyzes are based on non-parametric multiple pairwise-comparison Wilcoxon rank sum tests using “BH” adjustment: * = *p* < 0.05, ** = *p* < 0.01, *** = *p* < 0.001 (Supplementary Table S6). Created with R (v4.2.2, x64)/R Studio (Version 2022.12.0 + 353)^45,46^.

VNTs specific for WNV, TBEV, and USUV were employed to differentiate between flavivirus antibodies detected by the commercial ELISA. Figure 3 illustrates the distribution (a–c) and the levels of nAb titers (d–f) against the various flaviviruses for each species. Of the 61 dog sera positive in the flavivirus-ELISA, 56 samples could be further differentiated using the specific VNTs. Similarly of the 794 wild boar sera positive for flavivirus Abs a subset of 145 samples were exemplarily differentiated using the VNTs. Finally, of the 26 flavivirus-positive small ruminant sera 24 were further differentiated. In all three tested animal categories WNV-, USUV-, and TBEV-specific nAbs could be found. The percentage of WNV and TBEV nAbs was similar in the dogs (32.14%; 95% CI 20.29–45.96 and 30.36%; 95% CI 18.78–44.10, respectively) and higher than for USUV (7.14%; 95% CI 1.98–17.29). This was also observed in the statistically higher antibody titers against WNV and TBEV than against USUV (Wilcoxon rank sum test: *p* < 0.05* and *p* < 0.01**, respectively). Of the wild boar samples, WNV nAbs were most prevalent (28.28%; 95% CI 21.12–36.35) followed by TBEV (15.86%; 95% CI: 10.33–22.84) and USUV (14.48%; 95% CI 9.19–21.28) with statistically higher antibody titers against WNV than USUV (Wilcoxon rank sum test: *p* < 0.05*). Yet interestingly a large subset of the wild boar samples (41.38%; 95% CI 33.27–49.85) could not be allocated to a specific flavivirus. Lastly, the majority of the nAbs from the small ruminant sera was specifically against TBEV (41.67%; 95% CI 22.11–63.36), these also producing statistically higher antibody titers than against USUV (Wilcoxon rank sum test: *p* < 0.05*). Supplementary Table S5 summarizes the results from the three VNTs: distribution of nAbs and the resultant seroprevalences. Supplementary Figure S1a–f shows the larger extent of cross-reactivity or past exposures to multiple flaviviruses in the wild boars than in the dogs, where seropositivity could be more easily allocated to one specific virus.

####  In-house NS1-ELISA

**Fig. 4:**
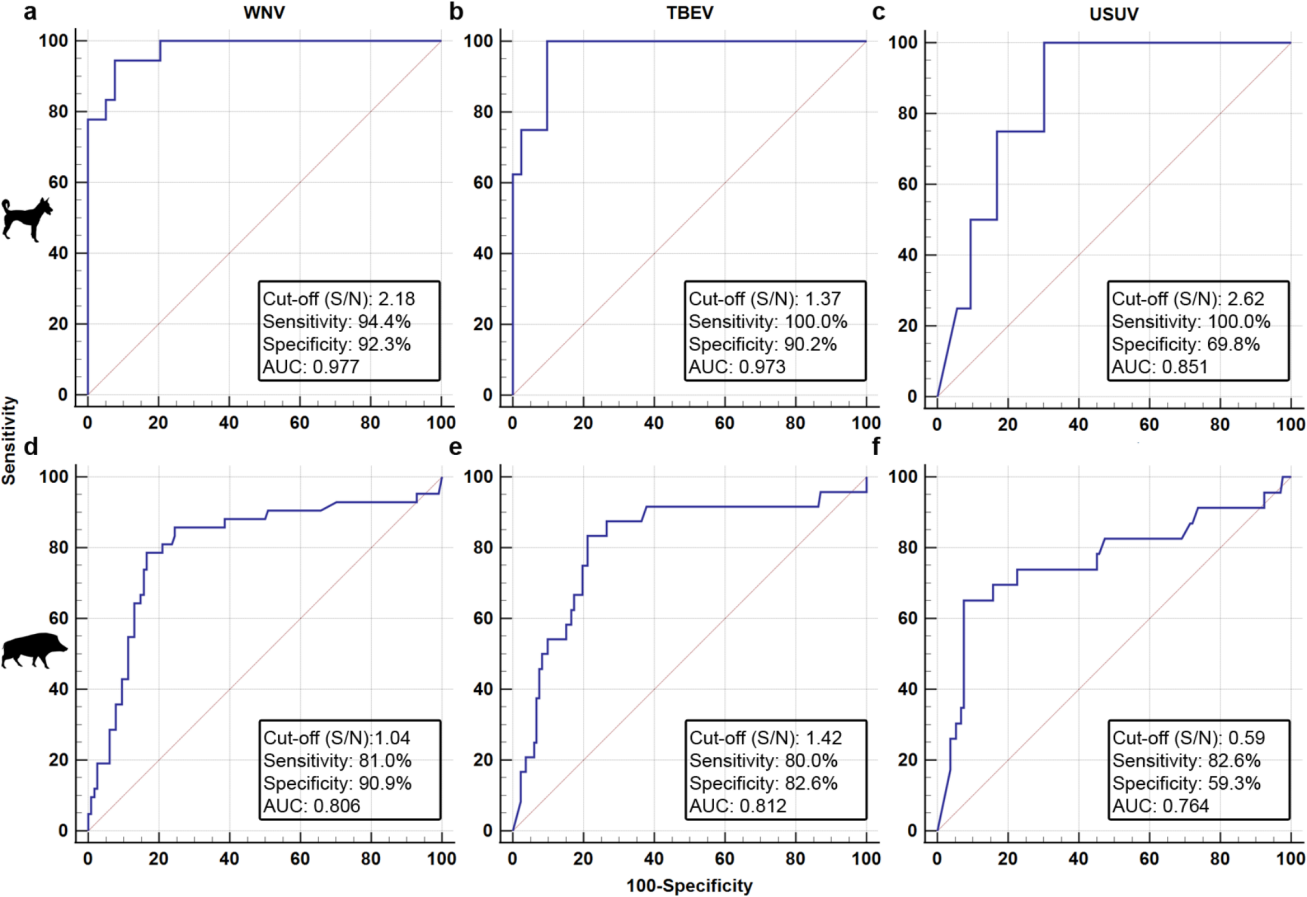
Validation of the in-house NS1-ELISA for detecting West Nile virus (WNV), tick-borne encephalitis virus (TBEV), and Usutu virus (USUV) specific antibodies in dog sera (*n* = 56; **a**–**c**) as well as wild boar sera (*n* = 145; **d**–**f**) based on the VNT-results as a gold standard. Receiver operating characteristic (ROC) curves depicting the area under the curve (AUC; shown by the blue line) for the NS1-ELISA as well as the estimated cut-offs. The statistical assessment of the performance of the NS1-ELISA is summarized in Supplementary Table S8.

An NS1-ELISA was validated based on the VNT results as a gold standard for the dog (Fig. 4a–c) and wild boar sera (Fig. 4d–f). All statistical parameters are summarized in Supplementary Table S8. Due to the small sample size of flavivirus-positive sera from small ruminants, ROC-analyzes were not conducted for these samples. The NS1-ELISA efficiently detected WNV and TBEV-specific sera, that had previously been confirmed by the VNTs to possess specific nAbs, in the dogs with a sensitivity and specificity of 94.44 and 92.31%, respectively for WNV and 100.00 and 90.24%, respectively for TBEV. For the wild boar sera, the ELISA parameters (sensitivity/specificity) were lower and slightly lower: 81.0 and 90.9%, respectively for WNV and 80.0 and 82.6%, respectively for TBEV.

**Fig. 5:**
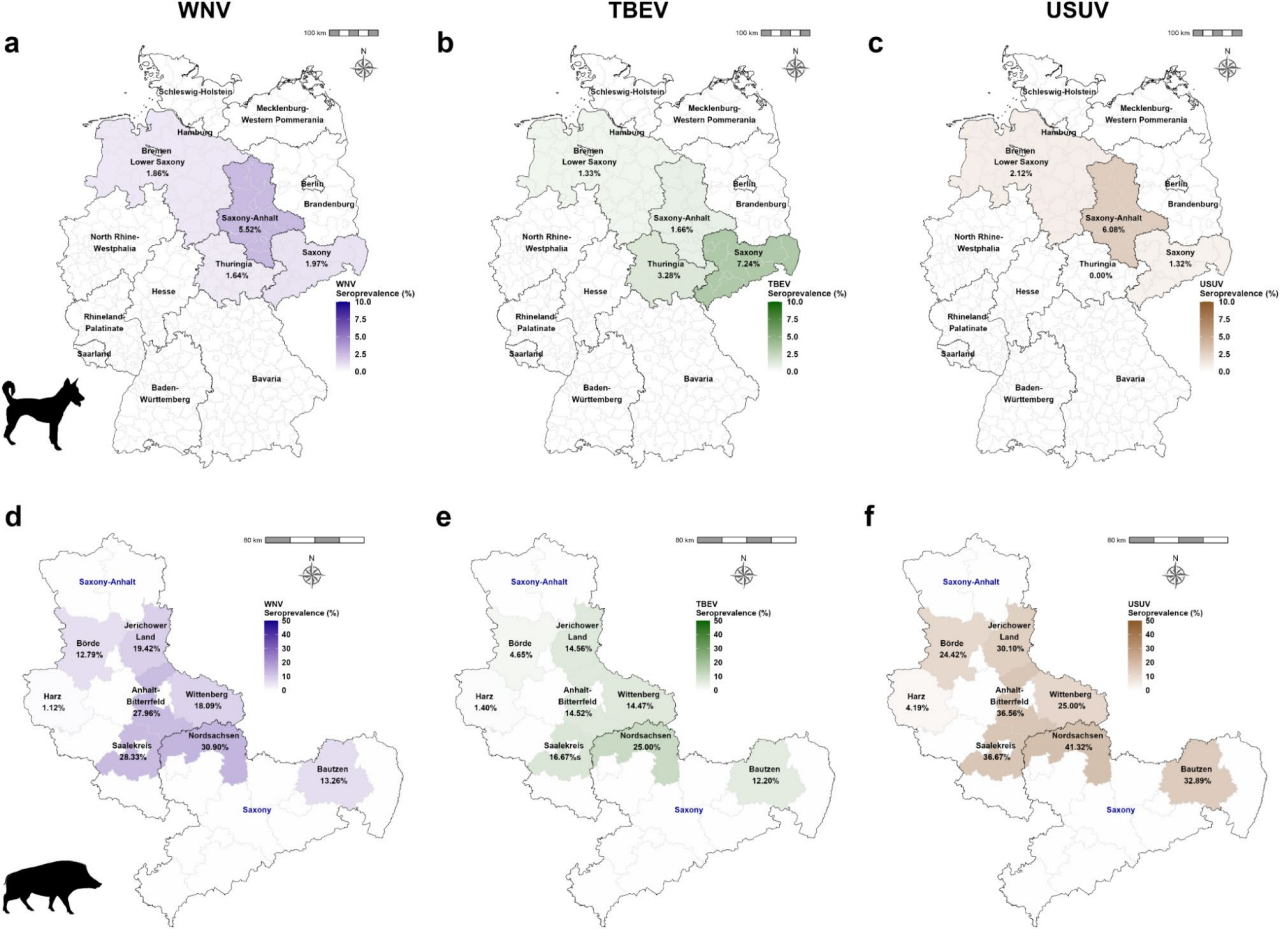
Seroprevalence of (**a**, **d**) West Nile virus (WNV), (**b**, **e**) tick-borne encephalitis virus (TBEV), and (**c**, **f**) Usutu virus (USUV) specific antibodies as detected by the in-house NS1-ELISA in wild boar and dog sera, respectively. Wild boar sera from the districts Saxony and Saxony-Anhalt were tested and results are depicted per district. Dog sera were tested from Saxony, Saxony-Anhalt, Lower Saxony, and Thuringia and due to the low sample size of flavivirus-positive small ruminant sera ROC-analyzes could not be performed and cut-offs not determined. Raw data and seroprevalences from the NS1-ELISA are listed in Supplementary Tables S1–S4; S7. Created with R (v4.2.2, x64)/R Studio (Version 2022.12.0 + 353)^45,46^. Map was created with GADM version 4.1. (https://gadm.org).

The determined cut-offs for WNV, TBEV, and USUV in the dog and wild boar sera were subsequently used to differentiate the detected flavivirus antibodies in the pre-screened samples. Calculated seroprevalences are depicted in the maps in Fig. 5 and in Supplementary Table S7. Raw data is listed in Supplementary Tables S1–S4. Of the 794 wild boar samples that had been tested positive for flavivirus nAbs by VNTs, 789 could be further evaluated using the NS1-ELISAs. The tests yielded very high seroprevalences for WNV followed by TBEV in the majority of the examined districts with Nordsachsen being the most affected (WNV: 30.90%; 95% CI 25.61–36.59 and TBEV: 25.00%; 95% CI 20.11–30.42). The district Harz had the lowest seroprevalences with only 1.12% (95% CI 0.31–2.84) for WNV and 1.40% (95% CI 0.46–3.23) for TBEV, followed by Börde with 12.79% (95% CI 6.56–21.73) for WNV and 4.65% (95% CI 1.28–11.48) for TBEV. Fewer flavivirus nAb-positive dog samples (*n* = 56) were available to evaluate the NS1-ELISA. Nonetheless, WNV and TBEV seroprevalences could be determined for the four tested federal states ranging from 1.64% (95% CI 0.04–8.80)–5.52% (95% CI 2.68–9.92) for WNV and 1.33% (95% CI 0.43–3.07)–7.24% (95% CI 3.67–12.60) for TBEV. In the case of WNV, ST was most affected whereas for TBEV it was SN (similar to the wild boars). The seroprevalences determined for USUV should be interpreted with caution as the specificities are very low in dogs (69.8%) and in wild boars (59.3%) (Fig. 4 and Supplementary Table S8). The NS1-ELISA could not be evaluated for the testing of small ruminant sera as not enough positive samples were found in the tested regions in eastern Germany.

## Discussion

### Deciphering flavivirus antibodies

The currently practiced monitoring of avian species and horses in combination with human findings can provide important information in the designation of endemic areas. The main objective of this study, however, was to accommodate the need for alternative sentinel species, readily available due to systematic serum banking, which can assist in detecting regions newly afflicted by WNV. The seropositivity of dogs, wild boar, and small ruminants was therefore verified for WNV, TBEV, and USUV in East and Central Germany. Particularly, wild boars and dogs appear to be ideal sentinel species in monitoring the circulation patterns of the three flaviviruses as they often seroconvert to high and stable titers after an infection without developing a detectable viremia. Nevertheless, it may be difficult to exclude travel-related infections in accordance with the movement of sampled dogs. As such, especially wild boars can be helpful prerequisites in the prediction of new WNV risk areas in the presence of assays that correctly distinguish between WNV, TBEV, and USUV antibodies in hunting material. In time of globalization, viral co-circulations are growing in importance and wild boars also appear to accurately reflect flavivirus co-circulations/infections in Germany, in endemic as well as not yet endemic areas. Lastly, the NS1-ELISA implemented in this study can be effectively used to differentiate between different flavivirus antibodies and specifically monitor WNV antibodies in wild boar and dog populations. Unlike VNTs, this technique does not require BSL-3 facilities, is more time- and cost-effective, and is less error prone when testing hemolytic samples obtained post mortem from wild boar carcasses. Future studies should monitor nationwide the seroprevalence of WNV in wild boars to further validate the species usage in detecting new virus incursions in non-endemic areas by means of the NS1-ELISA. However, it should be noted that the NS1-ELISA method has limitations, including lower sensitivity compared to other more complex diagnostic methods.

### Flavivirus seroprevalence in Germany — on a European level

The seroprevalence of flavivirus-specific Abs was examined in all three species and ranged from high in the wild boars (ST: 37.67%; 95% CI 38.14–43.98 and SN: 50.00%; 95% CI 46.14–53.86) to medium in the dogs (ST: 10.44%; 95% CI 6.40–15.82 and SN: 12.34%; 95% CI 7.59–18.59), and low in the small ruminants (ST/sheep: 1.83%; 95% CI 1.18–2.72 and SN/goat: 0.57%; 95% CI 0.07–2.03). The high seroprevalence in the wild boars (42.03%; 95% CI 39.79–44.30) is in accordance with the high occurrence of flavivirus Abs in the avifauna in central-eastern Germany (e.g., in 2019–2020 41.7%^6^). On a European level, higher flavivirus-seropositivity has only been described in wild boars from the south-east of Romania (63.24%)^57^. In general, however, the seroprevalences in wild boars from neighboring countries were distinctly lower: 4.1% in the Czech Republic^58^, 5.6% in France^59^, 7.7% in Italy^60^, 4%^15^ and 12.6% in Spain^61^, 17.6% in Serbia^62^, 17.1% in Belgium^63^, and 0% in Poland. The flavivirus-seroprevalences found in the dogs (7.86% (95% CI: 6.07–9.98)) in turn is similar to those found in horses from eastern Germany (including ST, SN, and Berlin/Brandenburg) from 2019 to 2021, where approximately 10% of the tested horses had flavivirus Abs^42,64,65^. Likewise they were similar to those found in dogs from neighboring countries such as Spain with 4.8%^35^ and France with 12.5%^34^ or 17.4%^66^. Seropositivity was higher in Denmark with 30.4%^33^ and Italy with 40.4%^67^. Lastly, in small ruminants, flavivirus surveillance studies are scarce and often focus solely on tick-borne encephalitis virus (TBEV), impeding the ability to make comparisons on a European level^63,68–71^.

### Flavivirus seroprevalences — regional variances

In the wild boars, the flavivirus seropositivity was not only statistically different when comparing the two central-eastern federal states (SN > ST) but differences were also observed on a district level: Saalekreis (63.39%; 95% CI 55.96–70.37) and Anhalt-Bitterfeld (56.15%; 95% CI 48.72–63.38) in the south-east bordering to SN were more affected than Harz (8.66%; 95% CI 5.96–12.07) in the north-west bordering to Lower Saxony (NI). Both sampled regions in SN had similarly high seropositivity (Nordsachsen: 51.04%; 95% CI 45.11–56.95 and Bautzen: 49.21%; 95% CI 4.08–54.36). The Saalekreis was also the region with the highest seropositivity in the sheep (6.52%; 95% CI 3.70–10.53) while no cases were detected in the north-western districts Harz and Börde. In the case of the dogs, the federal states of NI and Thuringia (TH) were also examined as states designated to be less or only very recently affected by WNV^6^. The seroprevalences were as expected statistically lower with 3.28% (95% CI 0.40–11.35) in TH and 5.54% (95% CI 3.46–8.35) in NI compared to 10.44% (95% CI 6.40–15.82) in ST and 12.34% (95% CI 7.59–18.59) in SN. This is consistent with findings from the avifauna, where even though WNV has, since 2018, steadily expanded to the west and south of central-eastern Germany^6^ a clear trend so far cannot be observed^10^. Nonetheless, indications for a westward expansion have been noted, as for example recent findings of WNV nAbs in racoon dogs from western NI^72^, and the first affected bird in the southern part of Hamburg^73^. TBEV has in the past also expanded from the south(-east) of Germany to the north-west, with multiple microfoci present in NI since a decade and recurrent human cases^74^. Lastly, since 2018, USUV is known to circulate throughout all federal states of Germany^9^.

### Deciphering antibody specificity to WNV, TBEV, and USUV

Genus specific flavivirus antibodies in the four mammalian species were differentiated by means of specific VNTs or NS1-ELISAs. As specific-nAbs were detected against all three viruses in all four species in the federal states of ST and SN, a continuous circulation of WNV, TBEV, and USUV can be assumed beyond the mosquito-mediated bird-to-bird and the tick-small mammal transmission cycle, respectively. All three viruses co-circulate not only geographically but also in susceptible hosts. Therefore, since wild boars appear to simultaneously develop nAbs against multiple different flaviviruses they can accurately reflect flavivirus transmission dynamics in Germany. In this study high antibody titers were often present in the wild boar sera against two or even three of the tested flaviviruses, as confirmed by the VNTs and NS1-ELISAs. Out of the 145 flavivirus-seropositive sera tested using the VNT, 60 (41.38%; 95% CI 33.27–49.85) could not be differentiated and were classified solely as “flavivirus” nAbs. These samples also accounted for a significant proportion of those that tested seropositive for two or all three flaviviruses in the NS1-ELISA (372 of 789; 47.15%; 95% CI 43.62–50.70). Exemplarily for this are five wild boars (WS/20/22 and WS/21/11, 119, 136, 312) from the east of ST which had antibody titers against all three viruses in the NS1-ELISAs and almost consistently high titers in the VNTs (ND_50_ WNV: 240–640; TBEV: 40–800; USUV: 40–240). Such high antibody titers against multiple flaviviruses are rather indicative for simultaneous or sequential infections than for serological cross-reactivity between closely-related flaviviruses, as has been described for several birds in Germany^75^. Lastly based on the VNTs, the wild boar flavivirus nAbs were more frequently specific to WNV than TBEV. While in the dogs the occurrences of WNV and TBEV nAbs were very similar and in the small ruminants TBEV nAbs dominated. USUV nAbs were least frequent in all of the tested species.

### WNV seroprevalence in Germany

The WNV seroprevalences (based on the NS1-ELISAs) in the two central-eastern federal states (ST and SN) were 17.64% (95% CI 15.94–19.44) in the wild boars and 3.90% (95% CI: 2.09–6.58) in the dogs. Three of the sheep from ST and one goat from SN were also reactive in the WNV VNT (ND_50_: 160, 80, 40, and 120, respectively). The detection of WNV antibodies in the wild boars and dogs from all of the examined districts nicely correlates with the detection of WNV nAbs in the avifauna^6,73^. Highly endemic districts for WNV, with annually reoccurring detections in birds and horses in the last five years (2018–2022)^73^, also had high seroprevalences in the wild boars, e.g., in Nordsachsen (30.90%; 95% CI 25.61–36.59) and Saalekreis (28.33%; 95% CI 21.88–35.52). Similarly, regions with WNV detections only twice in the last five years^73^ had significantly lower seroprevalences in the wild boars, e.g., in Bautzen (13.26%; 95% CI 10.00–17.11) and Börde (12.79%; 95% CI 6.56–21.73). There is also an especially good correlation between the collected data from the wild boars and observations made in horses from the same region of Germany^64,65^. In general, slightly lower WNV seropositivity in wild boars was described by colleagues from Serbia (WNV nAbs in 59% of the 17.6% that had flavivirus nAbs)^62^, Spain (WNV nAbs in 42.9% of the 12.6% that had flavivirus nAbs)^61^, and the Czech Republic (4.1%^76^ and 6.5%)^58^. By comparison, the seroprevalence of WNV in dogs was higher in other European countries. For example with 6.7–8.4% in France^34,37^, 36.9% in Serbia^77^, , 37.7% in Turkey^39^, and 55.5% in Italy^38^. It is of no surprise, that Italy has such a high seroprevalence in its stray dogs as the country repeatedly experiences high numbers of human as well as equine infections, as for example 723 and 47 cases in 2022, respectively^78^. It must also be kept in mind that all other European studies focused heavily on stray dogs kept in animal shelters or working dogs used in the military or for hunting. These dogs therefore spend the majority of their time outdoors and are more readily exposed to mosquitoes than the standard pet dog may be. Only in Spain, was the seroprevalence lower with 1.3–1.6%^35^ as well as in Belgium where none of the tested dogs carried WNV nAbs^36^. In Belgium, autochthonous WNV infections have not been noted to date even though neighboring countries such as the Netherlands have become recently affected^79^.

### TBEV seroprevalence in Germany

Human TBEV is a notifiable disease in Germany with high risk-areas situated in the southern federal states of Bavaria and Baden-Württemberg^74^. Since its introduction, TBEV has been expanding north-eastward and the districts Anhalt-Bitterfeld (ST) and Bautzen (SN) included in this study are nowadays also deemed as human TBEV risk-areas^74^. Hence it is not surprising that TBEV seroprevalences of 12.91% (95% CI 11.43–14.51) (ST: 10.27%; 95% CI 8.62–12.11 and SN: 17.74%; 95% CI 14.91–20.86) were observed in wild boars and 4.20% (95% CI 2.32–6.95) (ST: 1.66%; 95% CI 0.34–4.77 and SN: 7.24%; 95% CI 3.67–12.58) in dogs, with SN being more affected by TBEV. Likewise, fellow researchers had determined a TBEV-seroprevalence of 10.5% in wild animals (mainly wild boars) from SN from 2012 to 2013^80^ and 2.90% in wild boars from NI from 2019 to 2021^25^. In the frame of this study, TBEV nAbs were additionally found in wild boars from several districts that so far are not classified as risk areas: Nordsachsen, Börde, Harz, Jerichower Land, Saalekreis, and Wittenberg. Interestingly, also the two partial TBEV sequences from this study derived from wild boars shot in Saalekreis and Wittenberg, proving even recent virus transmissions in these two districts. The same is true for the dogs, where TBEV nAbs were found in non-endemic areas from eastern NI, including Hannover, yet with of course a lower seropositivity (1.33%; 95% CI 0.43–3.07) than in SN (7.24%; 95% CI: 3.67–12.58) and TH (3.28%; 95% CI 0.40–11.35). The study from Topp et al.^25^ describes a similar seroprevalence of 1.1% in dogs from NI and two other studies^81,82^ confirmed that natural foci of TBEV were already present in Hannover in the past. Nonetheless, TBEV seroprevalence in NI is still much lower than in the highly endemic southern part of Germany with 22.1%^83^. When comparing TBEV seroprevalences on a European level results varied greatly: in the Czech Republic with 11.3–13.4% (based on ELISA)^84,85^, in Norway with 16.4% (based on ELISA)^86^, in Belgium with 0.1% (based on VNT), in Denmark with 4.8% (based on VNT)^33^, and in Spain with 1.7% (based on VNT)^32^. An explanation for these variations in TBEV seroprevalence could be the usage of different serological assays, such as different commercial ELISA or VNT assays.

Small ruminants are also often mentioned in the context of TBEV due to a possible alimentary transmission of TBEV to humans through the consumption of raw milk products, as was recorded in different European countries (Austria, Estonia, Germany, Hungary, Poland, Slovakia, and The Czech Republic^87^). Small ruminants are known to develop only very low levels of viremia and readily seroconvert after a TBEV infection without portraying any clinical signs. As such sheep^88^ and goats^71,89–91^ have been used as useful sentinels in the past and Topp et al. found a TBEV seroprevalence of 0.4% in NI, Northern Germany. Similarly, in the here described study, only nine sheep (ST) and one goat (SN) carried TBEV-specific nAbs in East and Central Germany (seroprevalence of 0.69%; 95% CI 0.32–1.30 and 0.28%; 95% CI 0.01–1.57, respectively).

### USUV seroprevalence in Germany

Due to the lower sensitivity and specificity of the USUV-specific NS1-ELISA for the wild boar and dog sera the seroprevalences must be interpreted with caution and conclusions drawn are based on the results from the VNTs. USUV nAbs were measured in all three species, albeit at the lowest proportion and with the lowest median titers. The seroprevalence of USUV (based on VNT) in the dogs was 0.52% (95% CI 0.14–1.32), which was lower than the 13.1% found in hunting dogs in Italy^67^. The seroprevalence in the sheep was also 0.23% (95% CI 0.05–0.67) and since not all wild boar samples were examined via VNT the USUV seroprevalence cannot be inferred.

## Limitations of the study

Even though wild boars may be highly sensitive in monitoring WNV, TBEV, and USUV antibodies their usage is hampered by the fact that wild boars are frequently hunted in the winter, after the peak transmission season^62^. Wild boar surveillance, therefore, has its limitations in reflecting viral circulation during acute outbreaks throughout the summer season. Furthermore, future studies should focus on juvenile wild boars (after the subsiding of paternal immunity) in order to precisely detect recent viral exposures^15^. Surveillance data may be misinterpreted since adult wild boars appear to elicit higher WNV seroprevalences due to longer exposure times and a possible year-long persistence of nAbs (as verified in domestic pigs^92^)^59^. The quality of wild boar sera may also be poor due to hemolysis in accordance with post mortem blood collection. This in turn may lead to a higher number of false positives and restrictive cut-offs (e.g., 1:20 rather than 1:10 in VNTs^58^) should be set in the future taking the quality of each individual sera into account^93^. In the here described study, hemolysis appears to have had less of an effect on the interpretation of the NS1-ELISA results but rather on the qualitative evaluation of VNT plates from sera with antibody titers near to the detection limit. Finally, comparisons of seroprevalences among different studies must be interpreted with caution as often various diagnostic tests are used and the epidemic scenario in the different countries as well as the inclusion criteria among the different species may vary greatly (e.g., age, housing, and deployment outdoors)^35,84^.

## Electronic supplementary material

Below is the link to the electronic supplementary material.

Supplementary Material 1

Supplementary Material 2

## Data Availability

The data that supports the findings of this study are available in the main manuscript and the Supplementary Materials of this article. The two partial TBEV sequences have been deposited in GenBank (Accession nos. OR583627 and OR583628).
