# Supplementary material for: Tracking WNV transmission with a combined dog and wild boar surveillance system
Source: Sci Rep. 2025 Apr 1;15:11083. doi: 10.1038/s41598-025-89561-5 (PMC11962116; doi:10.1038/s41598-025-89561-5)
Supplement: Supplementary file 1 — Supplementary Material 1 [file 41598_2025_89561_MOESM1_ESM.pdf]

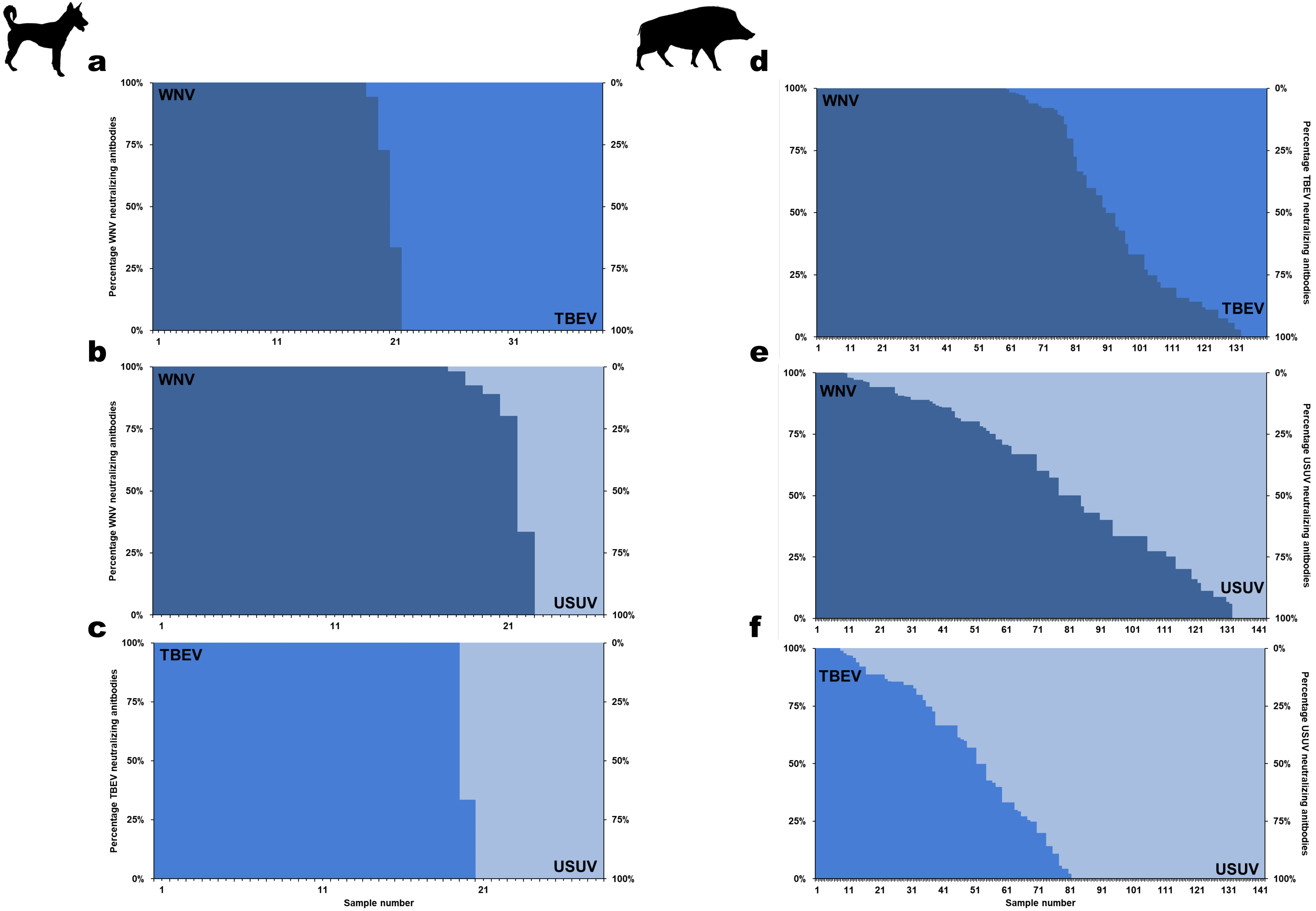

Figure S1. Bar charts depicting the percentage of neutralizing antibodies against two specific flaviviruses (WNV, TBEV, or USUV) in relation to one another for the dog (a-c) and the wild boar samples (d-f).
